# Supplementary material for: Role and mechanism of NCAPD3 in promoting malignant behaviors in gastric cancer
Source: Front Pharmacol. 2024 Apr 22;15:1341039. doi: 10.3389/fphar.2024.1341039 (PMC11070777; doi:10.3389/fphar.2024.1341039)
Supplement: Supplementary file 11 [file DataSheet2.ZIP › GSEA/immunologic/my_analysis.Gsea.1599462003817/GSE21063_CTRL_VS_ANTI_IGM_STIM_BCELL_NFATC1_KO_8H_UP.html]

Details for gene set GSE21063\_CTRL\_VS\_ANTI\_IGM\_STIM\_BCELL\_NFATC1\_KO\_8H\_UP[GSEA]

|  || Dataset | filtered\_dataset.sample\_info.cls#WT\_versus\_NCAPD3\_MUT |
| Phenotype | sample\_info.cls#WT\_versus\_NCAPD3\_MUT |
| Upregulated in class | NCAPD3\_MUT |
| GeneSet | GSE21063\_CTRL\_VS\_ANTI\_IGM\_STIM\_BCELL\_NFATC1\_KO\_8H\_UP |
| Enrichment Score (ES) | -0.593556 |
| Normalized Enrichment Score (NES) | -2.9299817 |
| Nominal p-value | 0.0 |
| FDR q-value | 0.0015751012 |
| FWER p-Value | 0.002 |
Table: GSEA Results Summary

  

Fig 1: Enrichment plot: GSE21063\_CTRL\_VS\_ANTI\_IGM\_STIM\_BCELL\_NFATC1\_KO\_8H\_UP      
 Profile of the Running ES Score & Positions of GeneSet Members on the Rank Ordered List

  

| SYMBOL | TITLE | RANK IN GENE LIST | RANK METRIC SCORE | RUNNING ES | CORE ENRICHMENT || 1 | 5295 | PIK3R1 | 467 | 0.493 | -0.3036 | No |
| 2 | 140688 | NOL4L | 495 | 0.475 | -0.2912 | No |
| 3 | 1119 | CHKA | 670 | 0.390 | -0.3905 | No |
| 4 | 22877 | MLXIP | 701 | 0.377 | -0.3869 | No |
| 5 | 55450 | CAMK2N1 | 861 | -0.269 | -0.4835 | No |
| 6 | 51646 | YPEL5 | 1014 | -0.413 | -0.5654 | Yes |
| 7 | 1408 | CRY2 | 1031 | -0.430 | -0.5481 | Yes |
| 8 | 11221 | DUSP10 | 1095 | -0.475 | -0.5617 | Yes |
| 9 | 1263 | PLK3 | 1134 | -0.502 | -0.5555 | Yes |
| 10 | 604 | BCL6 | 1136 | -0.504 | -0.5224 | Yes |
| 11 | 83548 | COG3 | 1211 | -0.588 | -0.5363 | Yes |
| 12 | 2043 | EPHA4 | 1228 | -0.602 | -0.5075 | Yes |
| 13 | 9975 | NR1D2 | 1230 | -0.605 | -0.4676 | Yes |
| 14 | 5187 | PER1 | 1254 | -0.641 | -0.4413 | Yes |
| 15 | 54520 | CCDC93 | 1260 | -0.647 | -0.4015 | Yes |
| 16 | 7994 | KAT6A | 1279 | -0.666 | -0.3698 | Yes |
| 17 | 202 | AIM1 | 1290 | -0.681 | -0.3313 | Yes |
| 18 | 80853 | KDM7A | 1311 | -0.714 | -0.2979 | Yes |
| 19 | 493861 | EID3 | 1315 | -0.729 | -0.2512 | Yes |
| 20 | 56605 | ERO1B | 1340 | -0.778 | -0.2163 | Yes |
| 21 | 113791 | PIK3IP1 | 1345 | -0.787 | -0.1664 | Yes |
| 22 | 27076 | LYPD3 | 1348 | -0.790 | -0.1149 | Yes |
| 23 | 6782 | HSPA13 | 1394 | -1.064 | -0.0760 | Yes |
| 24 | 51274 | KLF3 | 1406 | -1.296 | 0.0029 | Yes |
Table: GSEA details [plain text format]

  

Fig 2: GSE21063\_CTRL\_VS\_ANTI\_IGM\_STIM\_BCELL\_NFATC1\_KO\_8H\_UP      
 Blue-Pink O' Gram in the Space of the Analyzed GeneSet

  

Fig 3: GSE21063\_CTRL\_VS\_ANTI\_IGM\_STIM\_BCELL\_NFATC1\_KO\_8H\_UP: Random ES distribution      
 Gene set null distribution of ES for **GSE21063\_CTRL\_VS\_ANTI\_IGM\_STIM\_BCELL\_NFATC1\_KO\_8H\_UP**
